# Diet Quality and Comparison of Plant-Based Versus Omnivore Diets in Identical Twins: A Secondary Analysis of the Twins Nutrition Study (TwiNS)

**DOI:** 10.1016/j.cdnut.2025.107549

**Published:** 2025-09-04

**Authors:** Amanda B Zeitlin, Catherine P Ward, Alma Oralia Minerva Cooper, Matthew J Landry, Andrea M Krenek, Lindsay R Durand, Kristen M Cunanan, Jennifer L Robinson, Christopher C Dant, Christopher D Gardner

**Affiliations:** 1 Department of Pediatrics, Department of Medicine, School of Medicine, Stanford University, Palo Alto, CA, United States; 2 Stanford Prevention Research Center, Department of Medicine, School of Medicine, Stanford University, Palo Alto, CA, United States; 3 Department of Population Health and Disease Prevention, Joe C. Wen School of Population & Public Health, University of California, Irvine, Irvine, CA, United States; 4 Quantitative Sciences Unit, Department of Medicine, Stanford University, Palo Alto, CA, United States

**Keywords:** plant-based diet, vegan, dietary intervention, identical twins, diet quality, Healthy Eating Index

## Abstract

**Background:**

Omnivorous, vegan, and other diet patterns contain combinations of healthy and less-healthy foods. One aspect of equipoise in designing nutrition intervention studies is to emphasize high diet quality for all dietary patterns being contrasted.

**Objectives:**

This secondary analysis of an 8-wk long study was designed to qualitatively examine the alignment of participant diet assessment data with the original study design goal of achieving adherence to study diets that were both healthy and yet meaningfully different from one another.

**Methods:**

In this diet intervention, 22 pairs of identical twins were randomly assigned to a vegan or omnivorous diet and to consume either delivery-service meals (weeks 0–4) or prepare their own diet-appropriate meals/snacks (weeks 4–8). Data from 24-h dietary recalls at weeks 0, 4, and 8 were used to compare changes in intake of select food groups and nutrients. Linear mixed modeling evaluated changes in Healthy Eating Index-2015 (HEI) scores at weeks 4 and 8 compared with baseline, accounting for repeated measurements.

**Results:**

Both groups showed significant increase in their HEI scores during the study. Relative to baseline, mean changes in HEI total scores increased at 4 wk for both vegans (14.2) and omnivores (9.0), and these increases were largely maintained at 8 wk for both vegans (12.0) and omnivores (7.9). Healthy aspects similar for both groups included more vegetables and less added sugars. Differentiating factors included more legumes and fiber for vegans and more cholesterol and vitamin B-12 for omnivores.

**Conclusions:**

In this secondary analysis of a diet intervention trial, it is demonstrated that both the vegan and omnivore groups improved their diet quality during the study, while at the same time achieving substantive differences between the 2 groups in key nutrients/food groups. This allowed us to meaningfully contrast healthy versions of the 2 diets for their effects on previously reported health end points.

This trial was registered at clinicaltrials.gov as NCT05297825.

## Introduction

Chronic diseases such as diabetes, cancer, and cardiovascular disease (CVD) are the highest contributors to death, disability, and rising healthcare costs in the United States [1]. The Centers for Disease Control and Prevention estimates that 60% of all adults experience a chronic disease associated with poor diet quality [1]. Diets with greater amounts of whole foods, including vegetables, legumes, nuts and seeds, fruits, and whole intact grains are associated with fewer chronic diseases, whereas diets high in total and processed meat, refined grains, and added sugar increase risk of chronic disease [[2], [3], [4]]. High-quality diets (e.g., DASH, Mediterranean, or Havard’s Healthy Eating Plan) have been associated with a 12%–28% decreased risk of all-cause disease, CVD-related, and cancer-related morality [5]. Among such high-quality patterns, plant-based diets, which avoid or limit animal products [6], are associated with several positive health benefits, including reduced risk of CVD, coronary artery disease, ischemic heart disease, incidence of cancer, and decrease in risk factors such as high concentrations of LDL cholesterol, glucose, and BMI (in kg/m^2^) [[7], [8], [9], [10]].

A whole-food plant-based diet (WFPB), including vegan patterns, can differ significantly in nutritional quality from other dietary patterns [[11], [12], [13], [14]]. Compared with a typical American omnivorous diet, which consists of meat, fish, poultry, and eggs, a WFPB diet consists primarily of vegetables, legumes, nuts and seeds, fruits, and whole intact grains. A WFPB diet safely meets the USDA daily dietary requirements [15] with higher intakes of fiber, iron, potassium, and magnesium [16], providing a healthy, sustainable diet for all ages [17].

Although increasing intake of whole plant foods can improve nutrient intake, some plant-based meats are highly processed with increased saturated fats, sugars, and sodium and lower fiber [16]. Ultraprocessed foods, which are linked to increased weight and adverse health outcomes [18], have increasingly become a feature of plant-based diets, which can lead to varying health outcomes among individuals following different diet patterns [11,13]. Similarly, omnivorous diets can also vary in diet quality, which can produce differential effects on health [19,20]. Thus, in studies comparing plant-based with omnivorous diets, it is imperative to design both diets to be healthy to maximize equipoise and the value of result interpretation.

To this end, we undertook this secondary analysis of the Twins Nutrition Study (TwiNS) randomized trial to evaluate nutrition intake and diet quality in participants assigned to follow either a healthy vegan or healthy omnivorous diet. The primary analysis of TwiNS that focused on cardiometabolic outcomes was previously reported [21]. In this study, we provide a descriptive comparison of reported food group and nutrient changes over the study period, between and within study arms, to compare diet quality as an indicator of healthy food choices and demonstrate adherence to the primary study diet design.

## Methods

### Study description

**FIGURE 1:**
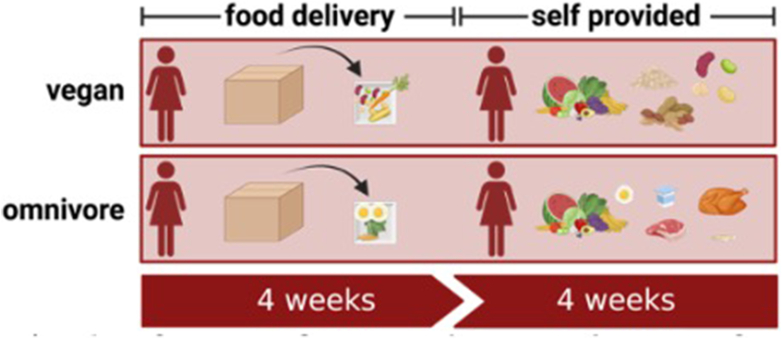
Study design. Reproduced from reference [21] with permission.

The purpose of the TwiNS RCT with parallel arms was to determine differences in the health outcomes of a healthy vegan diet compared with a healthy omnivorous diet with identical twins assigned to 1 of the 2 diets, aiming to control for genetic differences (Figure 1) [21]. The primary outcome was difference between diet groups in the 8-wk change from baseline in LDL cholesterol concentration [21], with secondary outcomes of cardiometabolic risk factors (e.g., plasma lipids, glucose, and insulin concentrations and serum trimethylamine N-oxide concentrations), plasma vitamin B-12 concentrations, and body weight. We also determined metrics of aging (i.e., telomeres and epigenetic clocks) [22] and the microbiome (manuscript under review). For this secondary analysis of TwiNS, our objective was to determine and compare nutrient and food group changes at major study time points among pairs of identical twins consuming a healthy vegan or healthy omnivorous diet. The sample size of 22 pairs of identical twins was determined based on resources rather than power calculations. A full description of the TwiNS methods was recently reported elsewhere [21].

Procedures for this study were followed in accordance with the ethical standards from the Helsinki Declaration and were approved by the Stanford University Human Subjects Committee (Institutional Review Board). All participants signed an informed written consent.

### Participants

The target population included 22 pairs of adult generally healthy identical twins aged 18 y or older. We excluded participants who weighed ≤45.4 kg (100 lb); had a BMI of ≥40, LDL cholesterol of ≥190 mg/dL, systolic blood pressure of ≥160 mm Hg, or diastolic blood pressure of ≥90 mm Hg; or who were pregnant or planning pregnancy. A complete list of study exclusion criteria can be found at https://clinicaltrials.gov (NCT05297825). Participants were recruited primarily from the Stanford Twin Registry between March and April of 2022. The first participant started the intervention in March 2022 and the last participant completed the intervention in July 2022.

### Randomization

After completing screeners to determine eligibility and consenting to participate in the study, a computerized random number generator was used to determine dietary assignment separately for each pair of twins. The generator was developed by a statistician who was not involved in data collection and executed by the study coordinator. Twins learned of their dietary assignment after completing all baseline measures and surveys.

### Dietary intervention

Over 8 wk, identical twins were randomly assigned to follow a healthy vegan diet (excluding all animal-sourced foods that included eggs, dairy, fish, and meat) or a healthy omnivorous diet comprising 60% healthy plant-based foods and 40% calories from optimal sources of eggs, dairy, fish, and meat (i.e., when accessible these were organic, pasture-raised, wild-caught, options). Both diets were designed to emphasize healthy patterns, such as increased vegetables and decreased added sugars and refined grains.

The 8-wk intervention was divided into 2 4-wk phases differing in food provision. During phase I, participants received fully prepared, calorie-controlled meals low in salt, added sugars, and saturated fat (breakfast, lunch, and dinner) delivered to their homes from Trifecta meal delivery service. Trifecta does not include dairy in their meals; therefore, both groups received prepared meals that were dairy free in phase I. Trifecta provided 119 different vegan meals and 121 different omnivorous meals, offering substantial variety. Supplemental Tables 1 and 2 show nutrient content and serving sizes of Trifecta meals participants received during the study. The study coordinator matched meals to the participants based on diet assignment and in accordance with any allergy restrictions. Participants were counseled to supplement the prepared meals with snacks to assist in reaching their individual daily intake targets for calories and food groups as outlined further.

During phase II, participants were asked to independently source and prepare all meals following guidelines from health educators over 3 virtual Zoom sessions at baseline and then at the beginning and middle of phase II, weeks 4 and 6 of the protocol. Participants also received email correspondence at weeks 1 and 7 with extra communications as needed from the health educators with resources for cooking and meal planning and the opportunity to ask questions about ways to maximize diet adherence. Health educators emphasized the importance of following a high-quality version of their respective diet assignments. Participants were instructed to eat high-quality diets by following 4 principles: *1*) eat minimally processed foods, *2*) meals should be well balanced consisting of vegetables, starch, protein, and healthy fats, *3*) a variety of foods should be consumed within each food group, and *4*) individual preferences should be considered. Vegan diet participants were asked to consume a daily average of ≥6 servings of vegetables, 3 servings of fruit, 5 servings of either legumes, nuts, seeds, or plant-based meat alternatives, and 6 servings of whole grains or starchy vegetables. Those assigned to the omnivore diet were asked to consume a daily average of ≥6–8 ounces of meat, fish, or poultry; ≤1 egg per day, 1.5 servings of dairy, 3 servings of vegetables; 2 fruits, 1 serving of legumes, nuts, or seeds; and 6 servings of whole grains or starchy vegetables.

### Dietary intake and quality assessment

Food intake was recorded by 3 unannounced phone calls (2 weekdays and 1 weekend; i.e., Saturday and Sunday) within a 1-wk window from trained nutrition professionals at baseline, 4 wk, and 8 wk. The complete 24-h dietary recalls were collected using Nutrition Data System for Research (NDS-R) (Nutrition Coordinating Center, 2021). Additionally, participants were instructed to keep food logs throughout the study using the Cronometer application (https://cronometer.com) to assist reporting food recalls and for health educators to provide feedback and guidance to participants during the study. Diet data collected from the NDS-R dietary recalls are reported in this study. Serving sizes for food groups were determined through use of the Nutrition Coordinating Center Food Group Serving Size Count System. For this analysis, we investigated 17 food categories and 20 nutrient values. See Supplemental Table 3 for specific components of our food groupings.

We assessed dietary quality by total and component Healthy Eating Index-2015 (HEI) scores calculated in NDS-R from the 9 dietary recalls collected at baseline, 4 wk, and 8 wk.

### Additional measures

Self-reported sociodemographic and anthropometric data on age, race, ethnicity, education, and weight were collected during enrollment. A detailed review of methodology and measures addressing cardiometabolic outcomes were recently reported elsewhere [21].

### Statistical analysis

Participants’ baseline characteristics are summarized as means (SDs) or percentages. For each timepoint (weeks 0, 4, and 8), the multiple dietary recalls that were collected to calculate HEI score were averaged for each participant to determine a single measure per participant per timepoint. The primary analysis includes all available data in a linear model with fixed effect for categorical time (weeks 0, 4, and 8) and a random effect to account for the correlated observations of each participant, stratified by diet type. Visual inspections were used to investigate model assumptions: normality of residuals and homogeneity of variance, using Q-Q plots and scatterplots, respectively. For our primary analysis, we present model estimates with 95% CIs for each dietary type. In a secondary analysis, we fit an additional linear mixed model with an interaction term of time by diet type, to evaluate a difference in the change of HEI score over time between the 2 diets: omnivore compared with vegan. Summary measures [means (SE)] are used to characterize specific nutrient and food groups at baseline, as well as, the changes at 4 and 8 wk from baseline, for each dietary type. Recall, these nutrient and food groups are used in part to calculate the HEI total scores. Data were analyzed using Stata version 17 (StataCorp) [23] and R version 4.4.2 [24].

## Results

### Baseline characteristics

We enrolled 22 pairs of twins (*N* = 44) randomly assigned to receive either a vegan or omnivorous diet (1 twin per diet): 21 pairs, with representation from both groups contributing to the final analyses. Most HEI scores (>95%) are based on all 3 planned recalls within a period. Participants were ∼40 y of age on average, mostly non-Hispanic White (72.7%) and females (77.3%), and generally healthy with a BMI of 25.9. Half of the twins reported being a college graduate (Table 1) [21].

**TABLE 1:** Baseline demographic and anthropometric characteristics.^1^

Characteristic	Vegan (*n* = 22)	Omnivore (*n* = 22)	All (*n* = 44)
Gender			
Female	17 (77.3)	17 (77.3)	34 (77.3)
Male	5 (22.7)	5 (22.7)	10 (22.7)
Age (y), mean (SD)	39.6 (12.7)	39.6 (12.7)	39.6 (12.7)
Highest level of education achieved			
High school graduate	0	2 (9.1)	2 (4.5)
Some college	7 (31.8)	5 (22.7)	12 (27.3)
College graduate	9 (40.9)	13 (59.1)	22 (50.0)
Some postgraduate school	2 (9.1)	0	2 (4.5)
Postgraduate degree	4 (18.2)	2 (9.1)	6 (13.6)
Race/ethnicity			
Asian	2 (9.1)	3 (13.6)	5 (11.4)
Black/African American	1 (4.5)	1 (4.5)	2 (4.5)
Native Hawaiian/PI	1 (4.5)	0	1 (2.3)
White	16 (72.7)	16 (72.7)	32 (72.7)
Multiracial	2 (9.1)	2 (9.1)	4 (9.1)
Weight (kg), mean (SD)			
Female	71.6 (12.9)	71.4 (12.1)	71.5 (12.5)
Male	68.7 (9.1)	72.7 (12.2)	70.7 (10.8)
Both sexes	70.9 (12.1)	71.7 (12.1)	71.3 (12.1)
BMI, mean (SD)			
Female	26.9 (5.0)	26.9 (4.9)	26.9 (4.9)
Male	22.6 (1.3)	23.0 (1.3)	22.8 (1.3)
Both sexes	25.9 (4.8)	26.0 (4.6)	25.9 (4.7)

Adapted from reference [21] with permission.

1 Values are n (%) unless specified.

Participant baseline diet data are summarized according to food categories in Table 2 and nutrients consumed in Table 3. Supplemental Figure 1 shows the main TwiNS participant CONSORT flow chart.

**TABLE 2:** Food category servings at baseline.^1^

Food categories	Vegan (*n* = 22)	Omnivore (*n* = 22)
	Mean (SE)	Mean (SE)
Vegetables	3.2 (0.3)	3.3 (0.4)
Fruit	1.2 (0.2)	1.2 (0.3)
Nuts and seeds	0.9 (0.3)	1.4 (0.4)
Legumes	0.2 (0.05)	0.3 (0.1)
Fat	2.5 (0.4)	2.3 (0.3)
Total animal protein	4.2 (0.4)	4.4 (0.5)
Eggs	0.4 (0.1)	0.5 (0.1)
Meat	1.9 (0.3)	1.9 (0.4)
Poultry	1.4 (0.3)	1.3 (0.3)
Fish	0.5 (0.2)	0.6 (0.2)
Total grain	6.0 (0.7)	5.4 (0.5)
Whole grain	1.5 (0.3)	1.0 (0.3)
Refined grain	4.3 (0.5)	4.1 (0.4)
Meat alternative	0.5 (0.2)	0.6 (0.3)
Dairy	1.4 (0.2)	1.6 (0.2)
Sweets	1.4 (0.3)	1.7 (0.5)
Sweetened beverages	0.4 (0.1)	0.2 (0.1)

1 Standard serving sizes based on Nutrition Coordinating Center NDSR Food Group Serving Count System. Food group servings sizes can be located in Appendix 10 of the NDSR manual, linked from the following resource: https://www.ncc.umn.edu/about-ncc/foods-nutrients-and-food-groups/. See Supplemental Table 3 for food groups classified within these food categories.

**TABLE 3:** Nutrient diet intake at baseline.

Nutrients	Vegan (*n* = 22)	Omnivore (*n* = 22)
	Mean (SE)	Mean (SE)
Energy (kcal)	1973 (131)	1931 (147)
Macronutrients		
Total fat (g)	85.7 (5.9)	85.7 (6.9)
%Calories from fat	38.0 (1.2)	38.8 (1.3)
Total saturated fat (g)	28.3 (2.1)	28.5 (2.3)
Total carbohydrates (g)	220.5 (16.2)	213.0 (19.0)
%Calories from carbohydrates	43.3 (1.1)	42.9 (1.2)
Starch (g)	103.1 (10.4)	93.2 (8.7)
Total Protein (g)	76.0 (5.3)	76.6 (6.2)
%Calories from protein	15.5 (0.7)	15.9 (0.8)
Animal protein (g)	45.9 (4.1)	44.9 (3.6)
Plant protein (g)	30.1 (3.1)	31.7 (3.8)
Fiber		
Total dietary fiber (g)	22.5 (2.0)	21.4 (2.2)
Soluble dietary fiber (g)	6.2 (0.5)	5.7 (0.6)
Insoluble dietary fiber (g)	16.1 (1.5)	15.5 (1.7)
Fiber (g) per 1000 calories	12.1 (1.1)	11.7 (1.0)
Sugars		
Total sugars (g)	75.8 (7.2)	79.8 (11.8)
Added sugars (g) (by total sugars)	42.8 (6.0)	47.7 (10.3)
Minerals		
Calcium (mg)	859.2 (77.8)	832.8 (65.5)
Iron (mg)	13.8 (1.3)	14.1 (1.4)
Sodium (mg)	3044 (284)	3130 (283)
Total folate (μg)	368.5 (34.7)	391.5 (36.2)
Vitamins		
Vitamin B-12 (cobalamin) (μg)	3.4 (0.3)	3.1 (0.3)
Fatty acids		
ω-3 Fatty acids (g)	2.3 (0.2)	2.4 (0.3)
Cholesterol (mg)	245 (25)	274 (28)

### Healthy Eating Index

**FIGURE 2:**
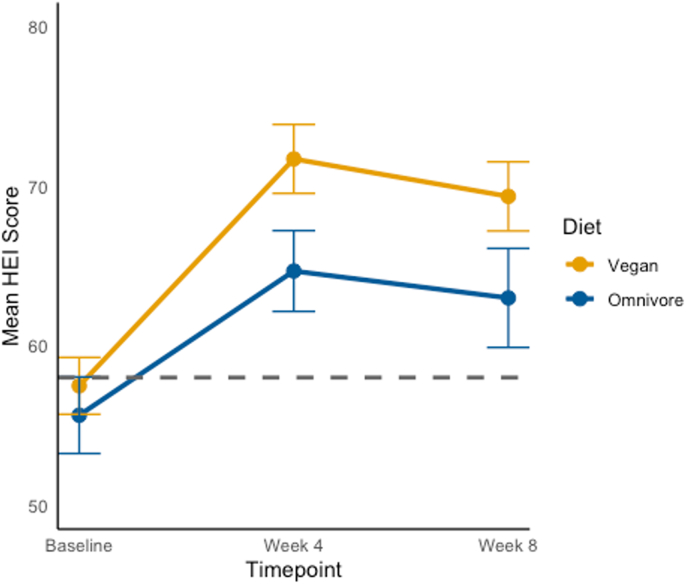
Healthy Eating Index total scores at baseline, 4 wk (end of phase I), and 8 wk (end of phase II). The mean Healthy Eating Index 2015 (HEI) total score for omnivores and vegans at baseline, week 4, and week 8, along with the standard error of the mean at each timepoint for each group. For both diets, as intended, the 4- and 8-wk HEI scores were significantly higher than baseline scores. In addition, at both week 4 and week 8, the HEI scores for vegans were higher than for omnivores. All findings *P* < 0.05. The gray dashed line represents the national average mean HEI total score, 58 [25].

The mean baseline HEI scores for the vegan and omnivore groups were 58.1 and 56.5, respectively. Compared with baseline, both groups showed significant increases in their HEI scores during the intervention (Figure 2) [25]. On average, participants on a vegan diet experienced HEI score increases of 14.2 (95% CI: 9.2, 19.3) at 4 wk from baseline and 12.0 (95% CI: 6.8, 17.1) at 8 wk from baseline. Those on an omnivore diet experienced a 9.0 (95% CI: 2.7, 15.4) increase in HEI score at 4 wk from baseline; a 7.9 (95% CI: 1.0, 14.6) increase at 8 wk from baseline. Supplemental Figure 2 presents a qualitative comparison of the component scores of the HEI.

Between-group HEI differences were tested using a linear mixed model evaluating an interaction term. We note, on average, participants on the omnivore diet achieved less of an increase in the change in HEI score at week 4 from baseline, compared with the vegan diet −5.1 (−13.2, 2.8); additionally, at week 8 compared with baseline, participants on the omnivore diet again achieved less of an increase in HEI score compared with vegan diet: −4.1 (−12.5,4.2). Model assumptions were reasonably met for all models.

### Dietary changes throughout the study intervention

**FIGURE 3:**
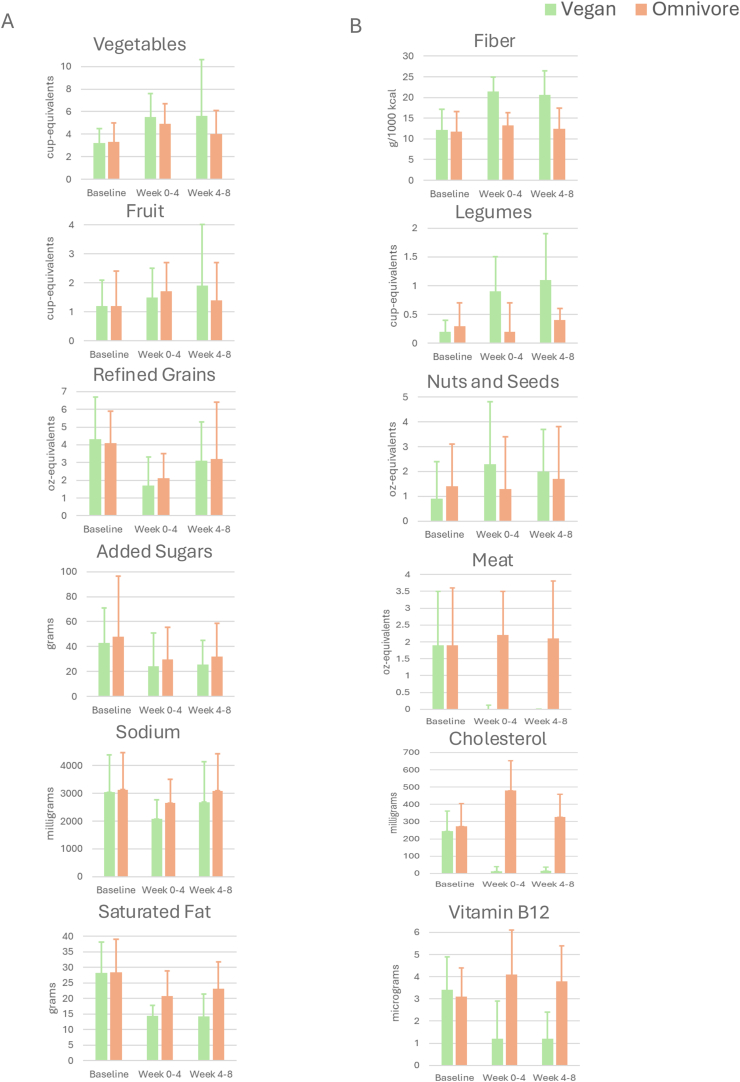
Select nutrient and food group changes during meal delivered and self-provided preparation of vegan and omnivore diets. Values are mean (SD) presented as absolute values of reported intake at the respective timepoint. (A) Food groups/nutrients that changed in optimal directions for both groups (increased vegetables, fruit; decreased refined grains, added sugars, sodium, and saturated fat). (B) Food groups/nutrients that differentiated vegan and omnivore groups (greater fiber, legumes, nuts and seeds consumption in the vegan group; greater meat, cholesterol, and vitamin B-12 intake in that omnivore group).

We analyzed the nutrient and food group changes of vegan and omnivore diets at week 4 and week 8 from baseline. The full set of changes are presented in TABLE 4, TABLE 5, TABLE 6, TABLE 7 and Supplemental Tables 4 and 5. A selection of the nutrients and foods groups are presented in Figure 3, with choices made that best illustrate the 2 objectives of diet design—*1*) making both diets healthy versions and *2*) making the 2 diets meaningfully different.

**TABLE 4:** Change in food category servings from baseline to week 4 (end of phase I).^1^

Food categories	Vegan (*n* = 22)	Omnivore (*n* = 22)
	Mean change (SE)	Mean change (SE)
Vegetables	2.3 (0.4)	1.7 (0.3)
Fruit	0.2 (0.2)	0.4 (0.3)
Nuts and seeds	1.4 (0.5)	−0.1 (0.4)
Legumes	1.0 (0.1)	0.1 (0.1)
Fat	1.5 (0.6)	2.5 (0.9)
Total animal protein	−4.1 (0.4)	3.2 (0.6)
Eggs	−0.4 (0.1)	0.9 (0.2)
Meat	−1.9 (0.3)	0.3 (0.5)
Poultry	−1.4 (0.3)	1.4 (0.4)
Fish	−0.5 (0.2)	0.6 (0.3)
Total grain	−2.1 (0.4)	−2.0 (0.6)
Whole grain	0.6 (0.3)	0.1 (0.3)
Refined grain	−2.5 (0.4)	−2.1 (0.5)
Meat alternative	3.4 (0.5)	−0.6 (0.3)
Dairy	−1.4 (0.2)	−0.7 (0.3)
Sweets	0.1 (0.4)	0.03 (0.6)
Sweetened beverages	−0.3 (0.1)	−0.2 (0.1)

1 Standard serving sizes based on Nutrition Coordinating Center NDSR Food Group Serving Count System. Food group servings sizes can be located in Appendix 10 of the NDSR manual, linked from the following resource: https://www.ncc.umn.edu/about-ncc/foods-nutrients-and-food-groups/. See Supplemental Table 3 for food groups classified within these food categories.

**TABLE 5:** Change in nutrient diet intake from baseline to week 4 (end of phase I).

Nutrients	Vegan (*n* = 22)	Omnivore (*n* = 22)
	Mean change (SE)	Mean change (SE)
Energy (kcal)	−346 (82)	−117 (136)
Macronutrients		
Total fat (g)	−15.6 (4.6)	−7.5 (6.1)
%Calories from fat	−0.4 (1.5)	−1.8 (1.6)
Total saturated fat (g)	−13.9 (1.8)	−7.8 (1.9)
Total carbohydrates (g)	−19.9 (11.5)	−30.4 (18.7)
%Calories from carbohydrates	4.1 (1.5)	−3.9 (1.7)
Starch (g)	−10.7 (8.0)	−17.9 (8.7)
Total protein (g)	−17.2 (3.9)	14.5 (6.6)
%Calories from protein	−2.9 (0.9)	4.8 (1.0)
Animal protein (g)	−44.8 (4.2)	20.8 (4.4)
Plant protein (g)	27.6 (2.8)	−6.3 (3.4)
Fiber		
Total dietary fiber (g)	12.0 (1.6)	2.1 (1.4)
Soluble dietary fiber (g)	2.4 (0.4)	0.2 (0.5)
Insoluble dietary fiber (g)	9.1 (1.3)	1.5 (1.1)
Fiber (g) per 1000 calories	9.4 (1.0)	1.6 (0.8)
Sugars		
Total sugars (g)	−18.5 (5.7)	−12.8 (12.0)
Added sugars (g) (by total sugars)	−18.5 (4.6)	−18.2 (10.3)
Minerals		
Calcium (mg)	−44.45 (58.8)	−138.1 (74.9)
Iron (mg)	6.0 (1.1)	0.3 (1.4)
Sodium (mg)	−967 (201)	−484 (301)
Total folate (μg)	129.7 (31.0)	−5.1 (28.5)
Vitamins		
Vitamin B-12 (cobalamin) (μg)	−2.1 (0.5)	1.0 (0.4)
Fatty acids		
ω-3 Fatty acids (g)	−0.5 (0.2)	−0.7 (0.3)
Cholesterol (mg)	−232 (24)	206 (46)

**TABLE 6:** Change in food category servings from baseline to week 8 (phase II).^1^

Food categories	Vegan (*n* = 21)	Omnivore (*n* = 22)
	Mean change (SE)	Mean change (SE)
Vegetables	2.3 (1.2)	0.8 (0.4)
Fruit	0.6 (0.5)	0.2 (0.3)
Nuts and seeds	1.1 (0.4)	0.2 (0.5)
Legumes	0.8 (0.2)	−0.1 (0.1)
Fat	1.4 (0.8)	1.4 (0.6)
Total animal protein	−4.1 (0.4)	1.7 (0.6)
Eggs	−0.4 (0.1)	0.2 (0.1)
Meat	−1.9 (0.4)	0.1 (0.4)
Poultry	−1.4 (0.3)	0.9 (0.4)
Fish	−0.4 (0.2)	0.4 (0.3)
Total grain	−1.2 (0.6)	−1.0 (0.5)
Whole grain	0.2 (0.4)	0.04 (0.3)
Refined grain	−1.3 (0.4)	−1.0 (0.6)
Meat alternative	2.9 (0.6)	−0.2 (0.3)
Dairy	−1.3 (0.2)	−0.1 (0.2)
Sweets	0.4 (0.3)	0.3 (0.7)
Sweetened beverages	−0.3 (0.2)	−0.01 (0.1)

1 Standard serving sizes based on Nutrition Coordinating Center NDSR Food Group Serving Count System. Food group servings sizes can be located in Appendix 10 of the NDSR manual, linked from the following resource: https://www.ncc.umn.edu/about-ncc/foods-nutrients-and-food-groups/. See Supplemental Table 3 for food groups classified within these food categories.

**TABLE 7:** Change in nutrient diet intake from baseline to week 8 (end of phase II).

Nutrients	Vegan (*n* = 21)	Omnivore (*n* = 22)
	Mean change (SE)	Mean change (SE)
Energy (kcal)	−338 (92)	−76 (100)
Macronutrients		
Total fat (g)	−19.3 (5.7)	−4.9 (4.7)
%Calories from fat	−2.8 (2.2)	−0.1 (1.6)
Total saturated fat (g)	−14.2 (1.9)	−5.4 (1.6)
Total carbohydrates (g)	−12.6 (14.6)	−26.7 (17.6)
%Calories from carbohydrates	6.2 (1.6)	−5.2 (1.6)
Starch (g)	−1.6 (8.9)	−15.7 (7.8)
Total Protein (g)	−20.9 (4.0)	15.9 (5.0)
%Calories from protein	−3.3 (1.0)	4.7 (1.2)
Animal protein (g)	−44.1 (4.6)	19.1 (4.4)
Plant protein (g)	23.1 (3.0)	−3.2 (2.2)
Fiber		
Total dietary fiber (g)	10.0 (2.5)	1.0 (2.0)
Soluble dietary fiber (g)	1.5 (0.5)	0.2 (0.5)
Insoluble dietary fiber (g)	8.3 (2.1)	0.6 (1.7)
Fiber (g) per 1000 calories	8.3 (1.7)	0.7 (1.0)
Sugars		
Total sugars (g)	−16.5 (7.3)	−9.4 (11.5)
Added sugars (g) (by total sugars)	−17.3 (5.7)	−15.7 (10.7)
Minerals		
Calcium (mg)	−103.4 (59.8)	36.1 (71.3)
Iron (mg)	2.3 (1.1)	−1.3 (1.2)
Sodium (mg)	−439 (215)	−36 (222)
Total folate (mcg)	110.9 (45.9)	−35.6 (27.0)
Vitamins		
Vitamin B-12 (cobalamin) (μg)	−2.2 (0.5)	0.6 (0.3)
Fatty acids		
ω-3 Fatty acids (g)	−0.3 (0.3)	−0.1 (0.3)
Cholesterol (mg)	−229 (27)	52 (32)

The data in column A of Figure 3 represent healthy messages given similarly to both diet groups, falling into the categories of what to emphasize and what to limit or avoid. It was recommended to both groups that they emphasize vegetables and fruits, and in both diet groups, these tended to be higher during both the food delivery phase and the self-provided phase. Likewise, it was recommended that both diet groups limit refined grains, added sugars, sodium and saturated fat, and in both diet groups, these tended to be lower during both the food delivery phase and the self-provided phase.

The data in column B of Figure 3 represent food groups and nutrients that would be expected to be different on a healthy vegan compared with a healthy omnivorous diet. As expected, fiber, legumes, and nuts and seeds tended to be higher at both the food delivery and the self-provided phase for the vegan group, whereas meat, cholesterol, and vitamin B-12 tended to be higher for the omnivore group.

Another overall trend in the data presented in Figure 3 is that the magnitude of the changes from baseline tended to be higher during the food delivery phase than during the self-provided phase. This was to be expected because the food delivery phase allowed for greater control over dietary intake. Although generally diminished during the self-provided meal phase, the changes from baseline and the comparisons between diet groups for the majority of the foods and nutrients presented in Figure 3 were observed to still be aligned with the intended directions of change.

#### Dietary changes—baseline to end of phase I

Other dietary changes presented in TABLE 4, TABLE 5 additionally demonstrated overall internal treatment fidelity (i.e., adherence) during the meal delivery phase of the study. By design, animal protein was omitted from the vegan diet with concomitant increases in plant protein and carbohydrate intake. Only vegans increased servings of meat alternatives compared with baseline. Vegans also reported decreased cholesterol and vitamin B-12 intake, whereas omnivores’ intake remained unchanged compared with baseline.

#### Dietary changes—phase I to phase II and baseline to end of phase II

Among the selected 17 food groups and 20 nutrient categories presented in Supplemental Tables 4 and 5 for changes between phase I and phase II, few differences within groups were observed, suggesting that the changes achieved during the meal delivery phase were mostly maintained. Given the similarities in intake of both groups between phase I and phase II, the differences observed from baseline to phase II (TABLE 6, TABLE 7) were similar to those from baseline to phase I (TABLE 4, TABLE 5).

## Discussion

In this secondary analysis of a dietary intervention trial of 22 pairs of generally healthy identical twins randomly assigned to eat either a healthy vegan or healthy omnivorous diet, we found that both vegan and omnivorous diets—as both delivered meals and self-prepared meals—significantly improved in quality throughout the study, with increased HEI scores observed for both groups at both timepoints compared with those at baseline. Two important objectives of our study design were to create healthy versions of both the vegan and the omnivorous diets yet design the 2 diets to still be sufficiently different that would allow for us to make a meaningful contrast between them. In addition to creating these healthy and different diets from a design perspective, it was also important to determine the extent of adherence to these strategies while we evaluated and implemented the study. Thus, we undertook this secondary analysis to explore in detail the participant reported dietary intake within study groups, for both the meal delivery and the self-provided phases, for a set of food groups and a separate set of nutrients. Our results support the successful implementation of the diet design objectives and thus support the internal validity of the study.

Similar to the average HEI score in the United States for population aged 2 y and older of 58 [25], the baseline scores for those assigned to the vegan diet and those assigned to omnivorous diet were 58.1 and 56.5, respectively. At the end of phase II, the vegan group had an average HEI score of 70.9, whereas the omnivores’ score was 62.9. Improvements in HEI among both groups are meaningful given that many studies have found higher HEI scores to be associated with positive health outcomes, including reduced long-term risk of CVD [26], diabetes [27], and some cancer-related mortality [28]. However, the greater increase in HEI scores found among the vegans is also meaningful given that a recent meta-analysis found a dose–response relationship between HEI-2015 and cancer-related mortality, CVD-related mortality and all-cause mortality. For instance, risk of CVD-related morality decreased by 0.51% per 1 score increment of HEI [29], indicating that a greater increase in diet quality can lead to additional positive health outcomes.

In phase I, during which participants ate the 3 prepared (delivered) meals daily, both vegans and omnivores increased their consumption of vegetables and decreased their consumption of total and refined grains, saturated fats, and sweetened beverages, changes that align with recommendations in the Dietary Guidelines for Americans [30]. The greater increase in plant protein was accompanied by increased sources of fiber and folate among the vegans compared with the omnivores as expected in well-planned plant-based diets [31]. With removal of animal foods in vegan participants’ diets, the decrease in vitamin B-12 and increase in iron we observed is consistent with other studies assessing the nutritional content of plant-based diets [12,16,[31], [32], [33], [34]]. The increase in iron intake in a healthy vegan diet has been estimated to be higher than other diets related to a high intake of green leafy vegetables, nuts, grains, and beans as sources of iron. However, some studies addressing iron concentrations among different dietary patterns found lower iron blood concentrations among vegans, perhaps due to differences in the bioavailability of iron in plant food compared with that in animal food [33,35].

The improvements in diet quality among the vegans we observed are consistent with other similar studies. Several studies found that the vegan diet had the highest diet quality scores (as measured by HEI or adapted HEI scales) compared with other dietary patterns and concluded that eliminating animal products was associated with better diet quality [12,36]. A systematic review comparing vegetarian diets with nonvegetarian diets similarly found that, in 9 of 12 studies assessed, vegans had better diet quality, indicated by 4.6–16.4 higher HEI 2010 scores than nonvegans [37], which is consistent with our findings.

Other intervention studies also reported similar acceptability and greater adherence for those assigned to a plant-based diet compared to those assigned to an omnivorous diet or control diet [[38], [39], [40]]. Some studies have shown that participants being aware of what they are eating and how it made them feel influenced their food choices and intake [41,42]. Therefore, the greater diet changes required of the vegans may have contributed to the vegan participants attending more closely to their food choices, whereas omnivore participants may have been less aware of dietary changes they made from baseline and therefore less likely to maintain the changes when preparing foods on their own. Several barriers in shifting to a plant-based diet have been cited in the literature including anticipating that preparing plant-based meals will be too complicated or expensive, the perception that dietary deficiencies in a plant-based diet brings health risks, the desire to continue enjoying eating meat, and avoiding the stigma and negative stereotypes associated with eating a plant-based diet [[43], [44], [45], [46]]. The successful dietary improvements made among the vegans during phase I and sustained during phase II may have in part been due to meal delivery support offered by the study design, which reduced some of barriers commonly associated with switching to a plant-based diet. Along with the health education sessions, phase I may have served as a model for the participants as how to eat a healthy vegan diet on their own without a meal delivery service during phase II. Similar to other studies that found cooking and food skills interventions led to positive outcomes for plant-based diet interventions [47], this study’s use of both prepared meals in the first 4 wk of the trial and educational support throughout the trial likely benefited participants by helping them understand how to prepare high-quality, plant-based meals.

Our study had several strengths of design and implementation. Enrolling identical twins was beneficial because most of the pairs were raised with and maintained relatively similar eating patterns before being randomly assigned to the 2 study arms, thereby minimizing the prestudy dietary variability between the 2 arms. We also used state-of-the-art NDS-R data containing information on both nutrients and food groups and achieved a high rate of 24-h recalls completed (98%). We also used a comprehensive set of comparisons (i.e., baseline compared with week 4, week 4 compared with week 8, and baseline compared with week 8), allowing us to compare both initial change and degree of maintenance of change, as well as HEI scores and components that provided us with a summary of overall diet quality comparisons. Finally, the initial 4-wk period of food delivery facilitated participants’ high adherence to the diet, whereas the latter 4 wk of self-provided foods increased generalizability.

Our study had some limitations. Because our study followed up participant diets over 8 wk only, sustainability of longer-term changes could not be determined. Given our small sample size, the study may have not been powered to determine differences in some outcomes. Moreover, our diet data likely included underreporting or misreporting, as is commonly experienced with self-reported diet assessments; however, there was no reason to believe that there were systematic differences in reporting between the 2 diet arms. Finally, because the majority of our participants were highly educated White females and participants choosing to enroll in a dietary study may have a stronger interest in nutrition and changing their diet, our findings may be less generalizable to other populations.

Future research should consider longer follow-up periods to determine the extent to which participants maintain dietary changes beyond 8 wk. Future investigations should also seek to evaluate participants’ experiences in transitioning to a plant-based diet to identify the barriers as well as the facilitators in making and sustaining dietary adjustments. This may be useful in developing future interventions and informing clinical practice and for public health efforts to motivate dietary change.

In conclusion, this secondary analysis of dietary data from the TwiNS trial provides meaningful insight on detailed nutritional composition of vegan and omnivorous diets, both designed with equipoise, to comprise high-quality foods with and without food delivery. In our study, the initial food delivery phase likely supported some of the beneficial changes in both diet patterns that were maintained through the self-provided phase in this study. Designing diets to contrast in this manner can make communication of the findings more relevant and easier to understand for both health professionals and the general public.

## Author contributions

The authors’ responsibilities were as follows – ABZ, MJL, CPW, CDG, JLR: designed the research; ABZ, MJL, CPW, CDG: conducted the research; ABZ, MJL, CPW, AOMC, LRD, KMC: analyzed the data; ABZ, MJL, AMK, CPW, AOMC, CCD, CDG: wrote the article; ABZ: had primary responsibility for the final content; and all authors: have read and approved the final manuscript.

## Data availability

Data described in the manuscript, code book, and analytic code will be made available upon request pending approval.

## Funding

This study was funded by the Vogt Foundation (to CDG) and by the National, Heart, Lung and Blood Institute (grant T32HL161270; to CPW). These supporting sources had no such involvement or restrictions regarding publication.

## Conflict of interest

The authors report no conflicts of interest.
