# Supplementary material for: Diet Quality and Comparison of Plant-Based Versus Omnivore Diets in Identical Twins: A Secondary Analysis of the Twins Nutrition Study (TwiNS)
Source: Curr Dev Nutr. 2025 Sep 4;9(10):107549. doi: 10.1016/j.cdnut.2025.107549 (PMC12513280; doi:10.1016/j.cdnut.2025.107549)
Supplement: Multimedia component 1 [file mmc1.docx]

Diet Quality and Comparison of Plant-Based Versus Omnivore Diets in Identical Twins: A Secondary Analysis of the Twins Nutrition Study (TwiNS)

Zeitlin, AB

**Supplemental Table 1: Serving sizes for food categories for Trifecta meals delivered to participants**^1,2^

| **Variable** | **Vegan** | **Omnivore** |
| --- | --- | --- |
| Vegetables | 1.8 (1.0) | 1.8 (0.6) |
| Fruit | 0.1 (0.2) | 0.04 (0.1) |
| Nuts and seeds | 0.2 (0.3) | 0.1 (0.2) |
| Legumes | 0.6 (0.7) | 0.2 (0.4) |
| Fat | 1.4 (1.0) | 1.5 (0.8) |
| Total animal protein | 0.03 (0.2) | 3.3 (0.9) |
| Eggs | 0.03 (0.2) | 0.4 (0.7) |
| Meat | 0 | 0.97 (1.5) |
| Poultry | 0 | 1.2 (1.5) |
| Fish | 0 | 0.8 (1.6) |
| Total grain | 0.8 (0.9) | 0.5 (0.5) |
| Whole grain | 0.7 (0.9) | 0.4 (0.5) |
| Refined grain | 0.1 (0.3) | 0.2 (0.3) |
| Meat alternative | 1.3 (1.62) | 0.1 (0.56) |
| Dairy | 0 (0) | 0 (0) |
| Sweets | 0.2 (0.5) | 0.2 (0.49) |
| Sweetened beverages | 0 (0) | 0 (0) |

^1^ Values are means (Standard Deviations) ^2^Standard serving sizes based on Nutrition Coordinating Center NDSR Food Group Serving Count System. Food group servings sizes can be located in Appendix 10 of the NDSR manual, linked from the following resource: https://www.ncc.umn.edu/about-ncc/foods-nutrients-and-food-groups/. See Supplemental Table 3 for food groups classified within these food categories.

**Supplemental Table 2: Nutrient content for Trifecta meals delivered to participants**^1^

| **Variable** | **Vegan (*n*=22)** | **Omnivore (*n*=22)** |
| --- | --- | --- |
| Energy (kcal) | 373 (51) | 408 (59) |
| *Macronutrients* |  |  |
| Total Fat (g)       %Calories from fat | 14.6 (4.3)  33.2 (8.5) | 16.7 (2.6)  37.4 (9.4) |
| Total Saturated Fat (g) | 2.9 (2.1) | 3.9 (1.5) |
| Total Carbohydrates (g)      %Calories from Carbohydrates | 47.5 (8.7)  50.6 (11.8) | 34.6 (12.3)  32.0 (9.8) |
| Starch (g) | 26.1 (6.3) | 18.2 (10.2) |
| Total Protein (g)    %Calories from Protein | 18.1 (6.1)  16.1 (4.7) | 31.1 (6.8)  30.5 (4.6) |
| Animal Protein (g) | 0.16 (1.2) | 24.6 (7.2) |
| Plant Protein (g) | 17.9 (5.9) | 6.5 (4.0) |
| *Fiber* |  |  |
| Total Dietary Fiber (g) | 9.9 (3.8) | 5.7 (2.5) |
| Soluble Dietary Fiber (g) | 2.1 (1) | 1.2 (0.7) |
| Insoluble Dietary Fiber (g) | 7.3 (3.2) | 4.2 (2.2) |
| Fiber (g) per 1000 Calories | 26.3 (9.2) | 13.8 (6.1) |
| *Sugars* |  |  |
| Total Sugars (g) | 8.4 (3.6) | 7 (3.0) |
| Added Sugars (g) (by total sugars) | 1.5 (2.4) | 1.5 (2.5) |
| *Minerals* |  |  |
| Calcium (mg) | 215 (136) | 128 (61) |
| Iron (mg) | 6.8 (3.3) | 4.6 (1.8) |
| Sodium (mg) | 432 (208) | 541 (140) |
| Total Folate (mcg) | 178 (106) | 104 (52) |
| *Vitamins* |  |  |
| Vitamin B-12 (cobalamin) (mcg) | 0.08 (0.3) | 1.6 (1.1) |
| *Fatty Acids* |  |  |
| Omega-3 Fatty Acids (g) | 0.4 (0.3) | 0.4 (0.5) |
| Cholesterol (mg) | 0 (0) | 162 (118) |

^1^Values are means (Standard Deviations)

**Supplemental Table 3: NDS-R food groupings**

| **Vegetables:**  Dark-green Vegetables VEG0100  Deep-yellow Vegetables VEG0200  Tomato VEG0300  Other Vegetables VEG0600  Other Starchy Vegetables VEG0450  Vegetable Juice VEG0500  Avocado and Similar FRU0500  (Excludes: White Potatoes VEG0400, Fried Vegetables VEG0900, Fried Potatoes VEG0800, Vegetable Based Savory Snack FMC0100) |
| --- |
| **Fruit:**  Fruit excluding Citrus Fruit FRU0400  Citrus Fruit FRU0300  Citrus Juice FRU0100  Fruit Juice excluding Citrus Juice FRU0200  (Excludes: Fruit-based Savory Snack FRU0700, Fried Fruits FRU0600) |
| **Nuts and Seeds:**  Nuts and Seeds MOF0500  Nut and Seed Butters MOF0600 |
| **Legumes:**  Legumes (cooked dried beans) VEG0700 |
| **Fat:**  Oil FOF0100  Butter and Other Animal Fats – Regular FAF0100  (Excludes: Margarine – Regular FMF0100, Margarine - Reduced Fat FMR0100, Shortening FSF0100, Butter and Other Animal Fats - Reduced Fat FAR0100) |
| **Whole Grains:**  Grains, Flour and Dry Mixes - Whole Grain GRW0100  Loaf-type Bread and Plain Rolls - Whole Grain GRW0200  Other Breads (quick breads, corn muffins, tortillas) - Whole Grain GRW0300  Crackers - Whole Grain GRW0400  Pasta - Whole Grain GRW0500  Ready-to-eat Cereal (not presweetened) - Whole Grain GRW0600  Ready-to-eat Cereal (presweetened) - Whole Grain GRW0700  Cakes, Cookies, Pies, Pastries, Danish, Doughnuts and Cobblers - Whole Grain GRW0800  Snack Bars - Whole Grain GRW1000  Snack Chips - Whole Grain GRW0900 |
| **Refined Grains:**  Grains, Flour and Dry Mixes - Refine Grain GRR0100  Loaf-type Bread and Plain Rolls - Refined Grain GRR0200  Other Breads (quick breads, corn muffins, tortillas) – Refined Grain GRR0300  Crackers - Refined Grain GRR0400  Pasta - Refined Grain GRR0500  Ready-to-eat Cereal (not presweetened) - Refined Grain GRR0600  Ready-to-eat Cereal (presweetened) - Refined Grain GRR0700  Cakes, Cookies, Pies, Pastries, Danish, Doughnuts and Cobblers - Refined Grain GRR0800  Snack Bars - Refined Grain GRR1000  Snack Chips - Refined Grain GRR0900 |
| **Grains Total:**  Whole Grains + Some Whole Grains + Refined Grains |
| **Dairy:** Dairy + Diary Artificial +Dairy Sweetened  Milk – Whole DMF0100  Milk - Reduced Fat DMR0100  Milk - Low Fat and Fat Free DML0100  Cheese - Full Fat DCF0100  Cheese - Reduced Fat DCR0100  Cheese - Low Fat and Fat Free DCL0100  Cream FCF0100  Cream - Reduced Fat FCR0100  Cream - Low Fat and Fat Free FCL0100  Yogurt - Unsweetened Whole Milk DYF0300  Yogurt - Unsweetened Low Fat DYR0300  Yogurt - Unsweetened Fat Free DYL0300  Yogurt - Artificially Sweetened Whole Milk DYF0200  Yogurt - Artificially Sweetened Low Fat DYR0200  Yogurt - Artificially Sweetened Fat Free DYL0200  Yogurt - Sweetened Whole Milk DYF0100  Yogurt - Sweetened Low Fat DYR0100  Yogurt - Sweetened Fat Free DYL0100  Frozen Dairy Dessert DOT0100  (Excludes: Artificially Sweetened Flavored Milk Beverage Powder without Non-fat Dry Milk MSC1100, Sweetened Flavored Milk Beverage Powder without Non-fat Dry Milk, Ready-to-drink Flavored Milk – Whole DMF0200, Ready-to-drink Flavored Milk - Reduced Fat DMR0200,  Ready-to-drink Flavored Milk - Low Fat and Fat Free DML0200, Sweetened Flavored Milk Beverage Powder with Non-fat Dry Milk DML0300, Dairy-based Sweetened Meal Replacement/Supplement DOT0500, Dairy-based Artificially Sweetened Meal Replacement/Supplement DOT0600, Dairy-based Unsweetened Sweetened Meal Replacement/Supplement DOT0900, Pudding and other Dairy Dessert DOT0300, Artificially Sweetened Pudding and Other Dairy Dessert DOT0400) |
| **Sweets:**  Sugar SWT0400  Syrup, Honey, Jam, Jelly, Preserves SWT0500  Sauces, Sweet – Regular SWT0700  Chocolate Candy SWT0100  Non-chocolate Candy SWT0200  Frosting or Glaze SWT0300 |
| **Sweetened Beverages:**  Sweetened Fruit Drinks BVS0300  Sweetened Soft Drinks BVS0400  Sweetened Tea BVS0500  Sweetened Coffee BVS0100 |
| **Meat:**  Beef MRF0100  Lean Beef MRL0100  Veal MRF0200  Lean Veal MRL0200  (Excludes Game MRF0500 and Organ Meats MOF0100)  Lamb MRF0300  Lean Lamb MRL0300  Fresh Pork MRF0400  Lean Fresh Pork MRL0400  Cured Pork MCF0200  Lean Cured Pork MCL0200  Lean Cold Cuts and Sausage MCL0100  Cold Cuts and Sausage MCF0100  Meat-based Savory Snack FMC0200 |
| **Poultry:**  Poultry MPF0100  Lean Poultry MPL0100  Fried Chicken - Commercial Entrées and Fast Food MPF0200 |
| **Fish:**  Fish - Fresh and Smoked MFF0100  Lean Fish - Fresh and Smoked MFL0100  Shellfish MSL0100  Fried Fish - Commercial Entrées and Fast Food MFF0200  Fried Shellfish - Commercial Entrées and Fast Food MSF0100 |
| **Eggs**:  Eggs MOF0300 |
| **Total Animal Protein:**  Meat + Poultry + Fish + Eggs |
| **Meat Alternatives:**  Meat Alternatives MOF0700 |

Abbreviation: NDS-R, Nutrition Data System for Research

**Supplemental Table 4. Change in food category servings from week 4 to week 8 (end of Phase I to end of Phase II)**^1^

|  | **Vegan (*n*=21)** | **Omnivore (*n*=22)** |
| --- | --- | --- |
| **Food Categories** | **mean change (SE)** | **mean change (SE)** |
| Vegetables | -0.04 (1.2) | -0.9 (0.4) |
| Fruit | 0.4 (0.5) | -0.2 (0.3) |
| Nuts and seeds | -0.4 (0.5) | 0.4 (0.6) |
| Legumes | -0.3 (0.2) | -0.2 (0.1) |
| Fat | -0.3 (1.1) | -1.1 (0.7) |
| Total animal protein | 0.1 (0.1) | -1.5 (0.5) |
| Eggs | -0.02 (0.03) | -0.7 (0.2) |
| Meat | -0.02 (0.02) | -0.1 (0.4) |
| Poultry | 0.01 (0.01) | -0.5 (0.5) |
| Fish | 0.1 (0.1) | -0.2 (0.3) |
| Total grain | 1.0 (0.6) | 1.0 (0.7) |
| Whole grain | -0.4 (0.4) | -0.1 (0.3) |
| Refined grain | 1.3 (0.5) | 1.1 (0.7) |
| Meat alternative | -0.6 (0.6) | 0.4 (0.2) |
| Dairy | 0.04 (0.03) | 0.7 (0.1) |
| Sweets | 0.2 (0.3) | 0.3 (0.5) |
| Sweetened beverages | 0.1 (0.1) | 0.1 (0.1) |

^1^Standard serving sizes based on Nutrition Coordinating Center NDSR Food Group Serving Count System. Food group servings sizes can be located in Appendix 10 of the NDSR manual, linked from the following resource: https://www.ncc.umn.edu/about-ncc/foods-nutrients-and-food-groups/. See Supplemental Table 3 for food groups classified within these food categories.

Abbreviation: SE, standard error

**Supplemental Table 5: Change in nutrient diet intake from week 4 to week 8 (end of Phase I to end of Phase II)**

|  | **Vegan** *(****n=*21)** | **Omnivore (*n*=22)** |
| --- | --- | --- |
| **Nutrients** | **mean change (SE)** | **mean change (SE)** |
| Energy (kcal) | 9 (75) | 41 (119) |
| *Macronutrients* |  |  |
| Total Fat (g)       %Calories from fat | -3.7 (5.4)  -2.4 (2.1) | 2.5 (6.2)  1.7 (1.7) |
| Total Saturated Fat (g) | -0.3 (1.5) | 2.4 (1.4) |
| Total Carbohydrates (g)   %Calories from Carbohydrates | 7.7 (14.2)  2.5 (2.1) | 3.7 (16.5)  -1.3 (1.5) |
| Starch (g) | 9.1 (8.6) | 2.2 (11.4) |
| Total Protein (g)    %Calories from Protein | -4.3 (3.1)  -0.7 (0.7) | 1.4 (5.8)  -0.2 (0.6) |
| Animal Protein (g) | 1.1 (0.6) | -1.7 (3.8) |
| Plant Protein (g) | -5.4 (3.0) | 3.2 (3.2) |
| *Fiber* |  |  |
| Total Dietary Fiber (g) | -2.2 (2.2) | -1.1 (2.1) |
| Soluble Dietary Fiber (g) | -0.9 (0.5) | -0.1 (0.6) |
| Insoluble Dietary Fiber (g) | -0.9 (2.1) | -0.9 (1.6) |
| Fiber (g) per 1000 Calories | -0.8 (1.5) | -0.8 (0.7) |
| *Sugars* |  |  |
| Total Sugars (g) | 2.3 (6.8) | 3.4 (5.1) |
| Added Sugars (g) (by total sugars) | 1.4 (4.5) | 2.4 (4.9) |
| *Minerals* |  |  |
| Calcium (mg) | -68.9 (58.6) | 174.2 (77.5) |
| Iron (mg) | -4.1(1.0) | -1.6 (1.0) |
| Sodium (mg) | 533 (224) | 448 (321) |
| Total Folate (mcg) | -23.3 (42.5) | -30.4 (31.6) |
| *Vitamins* |  |  |
| Vitamin B-12 (cobalamin) (mcg) | 0.04 (0.3) | -0.4 (0.3) |
| *Fatty Acids* |  |  |
| Omega-3 Fatty Acids (g) | 0.3 (0.3) | 0.6 (0.3) |
| Cholesterol (mg) | 2 (7) | -154 (40) |

Abbreviation: SE, standard error

Supplemental Figure 1: Consort participant flow chart


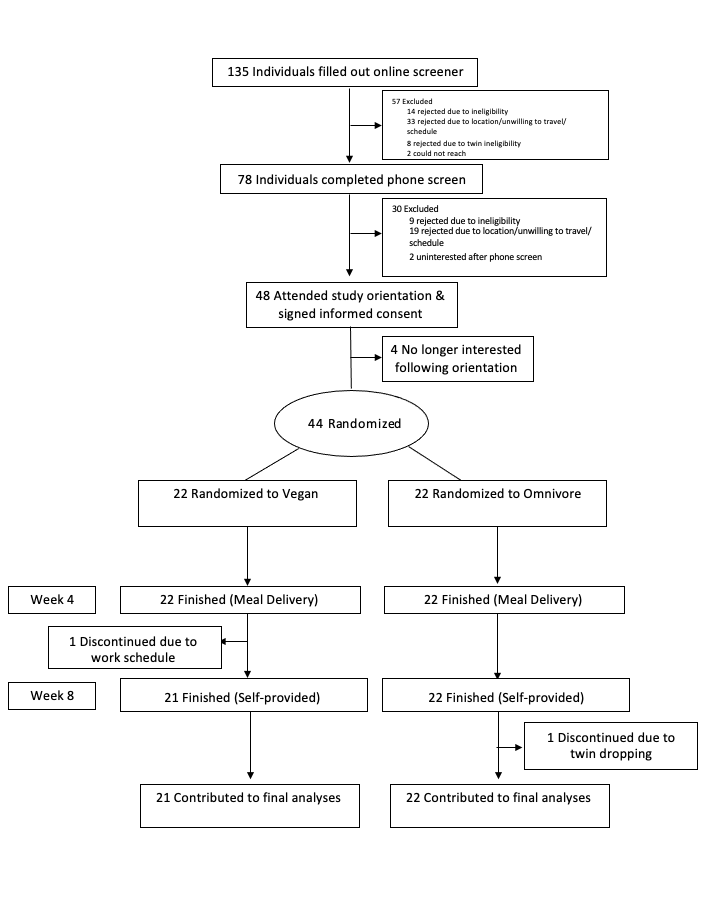


Reproduced from reference 21.**Supplemental Figure 2: Healthy Eating Index component scores at baseline, 4-weeks (end of Phase I), 8-weeks (end of Phase II)**


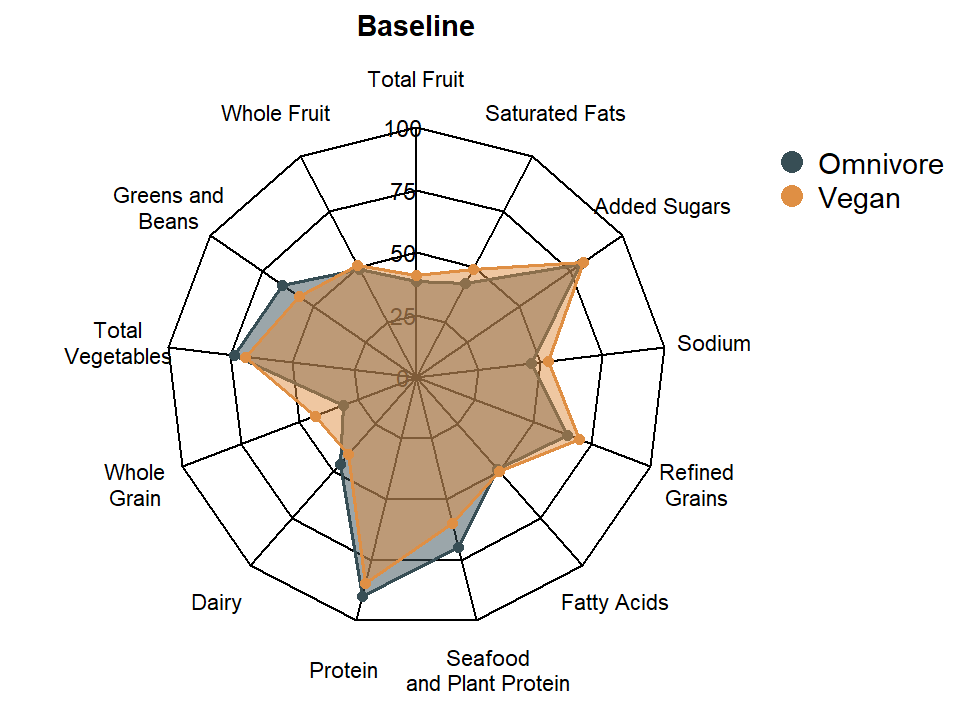

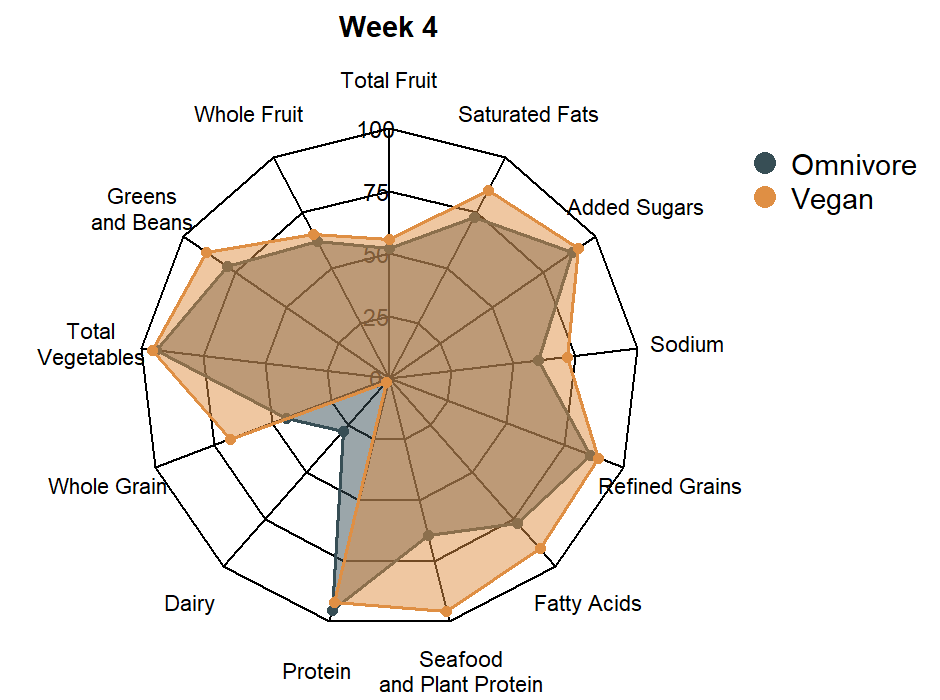


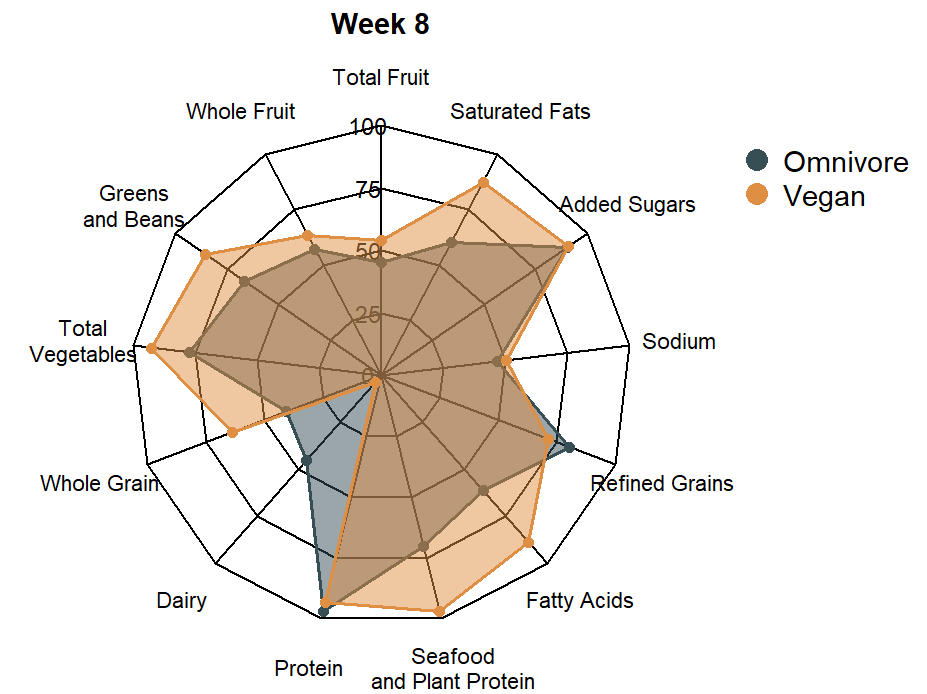


**Supplemental Figure 2: Healthy Eating Index component scores at baseline, 4-weeks (end of Phase I), 8-weeks (end of Phase II)**

**Figure S2** displays the mean HEI scores for the 13 HEI variables for vegans and omnivores. The radar plot is representative from 0-100 within each of the 13 categories. The maximum value within each category is represented by 100 and the minimum value within each category is represented by 0. The plots demonstrate the differences between vegan and omnivore groups at 0, 4, and 8 weeks of the study.
